# Exploring the Relationship Between CD 166 Expression and Breast Imaging Reporting and Data System (BI-RADS) Scores in Breast Cancer Patients and Healthy Volunteers

**DOI:** 10.7759/cureus.48145

**Published:** 2023-11-02

**Authors:** Vemareddy Hemalatha, Bhawna Dev, Nagasubramanian Vanitha Rani, Rajendran SD, Shabna Roupal

**Affiliations:** 1 Faculty of Pharmacy, Sri Ramachandra Institute of Higher Education and Research, Chennai, IND; 2 Department of Radiology, Sri Ramachandra Institute of Higher Education and Research, Chennai, IND; 3 Pharmacy Practice, Jaya College of Arts and Science College of Pharmacy, Chennai, IND; 4 Pharmacy, Scitus Pharma Services, Chennai, IND

**Keywords:** active leukocyte cell adhesion molecule (alcam), breast imaging reporting and data system (bi-rads), diagnosis, breast cancer, cd166

## Abstract

Background: This research embarked on a crucial endeavor to clarify the connection between levels of CD166 expression and the established Breast Imaging Reporting and Data System (BI-RADS) grading system. Through a comprehensive exploration of this correlation, the objective was to ascertain if CD166 could function as an additional biomarker, enhancing the predictive effectiveness of the BI-RADS classification.

Method: This prospective observational study involved 81 women with histopathologically confirmed early breast tumors and 81 radiologically confirmed healthy breast volunteers. The BI-RADS scores of all the participants included in the study were recorded. Before starting treatment, serum, saliva, and urine samples were collected. The CD166 levels were quantified using an enzyme-linked immunosorbent assay.

Results: The study involved the analysis and comparison of the mean and standard deviations of CD166 expression in serum, saliva, and urine across various BI-RADS categories. Notably, statistically significant differentiation was found (p=0.00) across all samples spanning the spectrum of BI-RADS categories.

Conclusion: A progressive rise in CD166 concentration coincides with the increasing gradient of the BI-RADS category, implying a possible link between CD166 and breast cancer progression and severity.

## Introduction

Breast cancer (12.5%) remains a formidable global health issue, affecting millions of women globally. For effective treatment planning and patient outcomes, early detection and precise risk assessment are critical [1]. According to the latest statistics on breast cancer in India, the estimated number of new cases in 2018 in India, is 1,62,468 (27.7%), and mortality is 87,090 (23.5%) [2]. The data obtained from different breast cancer registries across the country reflects the incidence varying from 30.7% in Chennai to 19% in Dibrugarh [3].

The Breast Imaging Reporting and Data System (BI-RADS) (5th Edition BI-RADS Atlas) is a standardized classification system for mammography findings and determining breast cancer risk [4-5]. The detailed categories of BI-RADS are mentioned in Table 1 [6]. While BI-RADS is useful, there is a rising interest in investigating new biomarkers that could supplement established diagnostic techniques and improve prognosis accuracy.

Cluster differentiation (CD166), also known as active leukocyte cell adhesion molecule (ALCAM), is a transmembrane glycoprotein that is involved in a variety of biological functions such as cell adhesion, migration, and signaling [7]. CD166 appears to be implicated in cancer progression and metastasis, making it a possible candidate for detecting high-risk breast cancer patients [8-9].

The purpose of this study is to look into the relationship between CD166 expression levels and the BI-RADS grading system in breast cancer patients. By investigating the relationship between CD166 and BI-RADS, we hope to find out whether CD166 can be used as a valuable adjunct biomarker to improve the predictive capacity of the BI-RADS classification, ultimately assisting in early diagnosis, risk stratification, and personalized treatment approaches for breast cancer patients.

Our work intends to shed light on the possible significance of CD166 in breast cancer diagnosis furthering our understanding of the disease and potentially paving the way for improved patient care and outcomes.

## Materials and methods

This nested case-control study was conducted in an 1800-bed tertiary care hospital. The study was approved by the institutional ethics committee (IEC No: IEC-NI/20/FEB/74/24) and informed consent was taken before the recruitment of the patient.

Sample size: With an expected sensitivity of 70%, with a precision of 10, and at a 95% confidence interval, the minimum sample size required was 81 female subjects confirmed with breast cancer (case group) and 81 healthy volunteers (control group).

Case group: A total number of 81 women with histopathologically proven malignant early breast tumors up to 3 cm in size were included.

Control group: A total number of 81 healthy women volunteers with no evidence of any health issues or breast abnormalities, as confirmed by mammography or breast ultrasound, were included in the control group.

Samples collection: A 3 ml of whole blood was collected in a red vacutainer tube and allowed for clotting for an hour. Patients were instructed to perform mouth rinsing before collecting the saliva sample. They were then asked to sit with their head tilted downward and collected unstimulated saliva in a sterilized Eppendorf tube. A total of 10 ml of urine was collected in a sterilized urine container. All the samples (serum, saliva, and urine) from included study participants cool centrifuged at 4°C and the supernatant was stored at -80°C.

To ensure the baseline assessment of CD166 expression from the case group, all the samples (serum, saliva, and urine) were obtained before the commencement of any treatment, such as neo-adjuvant chemotherapy or breast conservation surgery (BCS). From each study participant, relevant clinical data, including age, menopausal status, and any relevant medical history, were gathered for each participant. All included patients in the study with breast cancer had histopathological examinations, and their BI-RADS scores were calculated based on mammography findings, and control patients were also assigned BI-RADS scores within the range of 1 to 3. BI-RADS values encompass a range from 0 to 6, with higher scores indicating a higher likelihood of cancer. These scores were recorded by qualified radiologists who followed the Atlas 5th edition BI-RADS recommendations.

CD166/ALCAM expression analysis

The collected biological samples from breast cancer patients and healthy female volunteers were analyzed for ALCAM concentration by using a Human ALCAM ELISA kit (Abbkine, Korea). By using the enzyme-linked immunosorbent assay method, CD166 levels were quantified.

Statistical analysis

The collected data, including BI-RADS scores and CD166 expression levels, were entered into a statistical software package for analysis. Descriptive statistics, such as means, standard deviations, and frequencies, were calculated for demographic variables and BI-RADS scores. One-way ANOVA was used to assess the significant difference in CD166 expression in case and control groups.

## Results

In the present study, we evaluated CD166 levels in the serum, saliva, and urine of early breast cancer patients and healthy female volunteers. A total of 81 study participants were included in each group, the case group average mean age ± SD of patients was 49.93±10.9 and the control group average mean age ± SD of patients was 48.45 ±11.21. All the study participants were given BI-RADS scores based on the ACR Atlas 5th edition BI-RADS category by experienced radiologists (Table 1). Among the case group, a maximum number of patients, 54.3% (N = 44) had grade 2 tumors followed by grade 1, 24.7% (N=20) and grade 3 21% (N=17), and 93.8% (N = 76) had invasive mammary carcinoma, no special type (NST) type breast cancer, followed by ductal carcinoma in-situ 6.2% (N=5). Basic demographic details of the included study participants are mentioned in Table 2.

**Table 1:** ACR BI-RADS Atlas 5th Edition ACR: American College of Radiology; BI-RADS: The Breast Imaging Reporting and Data System [4,6]

BI-RADS Category	Impression		
0	Mammography: Incomplete – need additional imaging evaluation and/or prior mammograms for comparison ultrasound & MRI: Incomplete – need additional imaging evaluation		
1	Negative		
2	Benign (0%)		
3	Probably benign (<2%)		
4	Suspicious mammography and ultrasound	4A	Low suspicion of malignancy (2-10%)
		4B	Moderate suspicion of malignancy (10-50%)
		4C	High suspicion of malignancy (51-95%)
5	High suggestive of malignancy (>95%)		
6	Known biopsy-proven malignancy		

**Table 2:** Basic demographic details of included study participants SD: standard deviation; NST: no special type; N: no. of study participants

Demographics		Mean± SD or % (Case Group) N=81	Mean or % (Control Group) N=81
Age in years		49.93±10.9 (N=81)	48.45 ±11.21 (N=81)
Nottingham histologic grade of tumor	Grade 1	20 (24.7%)	-
	Grade 2	44 (54.3%)	
	Grade 3	17 (21%)	
Type of breast cancer	Invasive mammary carcinoma	76 (93.8%)	-
	NST ductal carcinoma in situ	5 (6.2%)	

When we conducted a comparative analysis between case and control group CD166 expression in serum, a statistically significant distinction was observed (p=0.000) as depicted in Table 3. Similarly, a notable statistical difference (p=0.000) was evident in Tables 4-5, when we juxtaposed CD166 expression between case and control groups in saliva and urine.

**Table 3:** Serum CD166 expression in different BI-RADS categories of included participants CD 166: cluster differentiation 166; BI-RADS: The Breast Imaging Reporting and Data System; N: no. of study participants; pg/ml: pico gram per ml; SD: standard deviation; F value: the ratio of between-group variation and within-group variation; P-value ≤0.05 considered as statistically significant

Sample	Group	BI-RADS category	N=162	Mean CD166 expression (pg/ml)	SD	F	P-value
Serum	Control group	1	45	36.9	11.9	37.5	0.000
		2	35	36.5	12.0		
		3	1	45.9	-		
	Case group	4	35	79.4	27.3		
		5	46	77.6	28.6		

**Table 4:** Saliva CD166 expression in different BI-RADS categories of included participants CD 166: cluster differentiation 166; BI-RADS: The Breast Imaging Reporting and Data System; N: no. of study participants; pg/ml: pico gram per ml; SD: standard deviation; F value: the ratio of between-group variation and within-group variation; P-value ≤0.05 considered as statistically significant

Sample	Group	BI-RADS category	N=162	Mean CD166 expression (pg/ml)	SD	F	P-value
Saliva	Control group	1	45	36.8	11.0	120	0.000
		2	35	32.8	10.5		
		3	1	28	-		
	Case group	4	35	86.6	19.0		
		5	46	82.8	15.7		

**Table 5:** Urine CD166 expression in different BI-RADS categories of included participants CD 166: cluster differentiation 166; BI-RADS: The Breast Imaging Reporting and Data System; N: no. of study participants; pg/ml: pico gram per ml; SD: standard deviation; F value: the ratio of between-group variation and within-group variation; P-value ≤0.05 considered as statistically significant

Sample	Group	BI-RADS category	N=162	Mean CD166 expression (pg/ml)	SD	F	P-value
Urine	Control group	1	45	42.3	11.3	47.7	0.000
		2	35	44.5	13.2		
		3	1	32.0	-		
	Case group	4	35	76.9	22.8		
		5	46	84.9	21.6		

**Figure 1:**
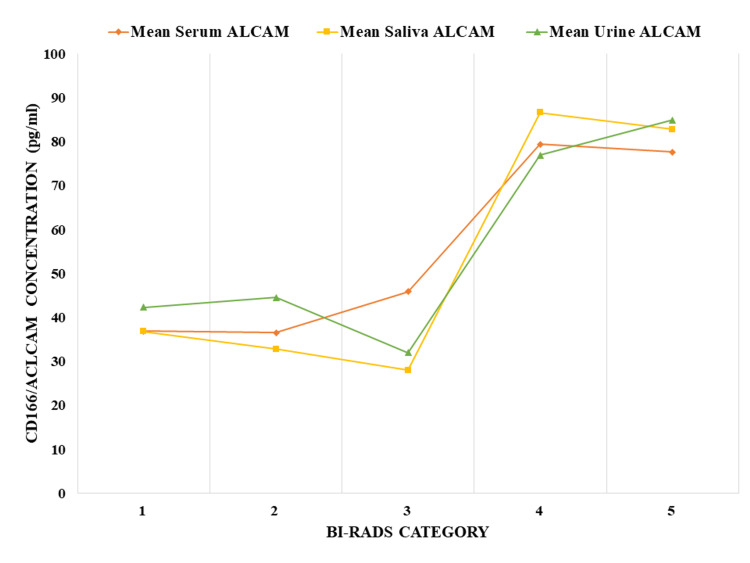
A line chart explains CD166 expression in different BI-RADS category patients CD166: cluster differentiation 166; ALCAM: activated leukocyte cell adhesion molecule; BI-RADS: The Breast Imaging Reporting and Data System; pg/ml: pico gram per ml

All the included patients were differentiated into groups based on their BI-RADS category (1-5) and mean and standard deviation are mentioned (Tables 3-5). Figure 1 graphically elucidates the progressive augmentation of CD166 concentration congruent with the ascending gradient of the BI-RADS category. This visual representation further reinforces the observed association between BI-RADS categorization and CD166 expression levels.

## Discussion

To the best of our knowledge and research, this is the first study that included 162 individuals to explore the relationship between BI-RADS categories, vs. serum, saliva, and urine CD166 expression, so currently this study is short for an original article. Previous investigations have demonstrated an upregulation of CD166 in breast cancer cases, highlighting a notable distinction in serum CD166 expression between breast cancer patients and healthy volunteers and concluded CD166 diagnostic accuracy [10-16]. Our findings revealed a significant and persuasive discrepancy (p=0.00) in the association between BI-RADS categories (1-5) and serum, saliva, and urine CD166 expression (Tables 3-5). These findings highlight the potential of CD166 expression in serum, saliva, and urine as relevant biomarkers for distinguishing differences across distinct BI-RADS categories. A gradual increase in CD166 concentration corresponds to the increasing gradient of the BI-RADS category, implying that CD166 may play a role in reflecting breast cancer development and severity.

The limitations and primary challenges in this study revolved around identifying individuals with early-stage breast cancer and acquiring biological samples upon immediate diagnosis without any treatment also this study was limited by its small sample size and the fact that it was conducted at a single center. While the collection of unstimulated saliva and processing of saliva samples pose some difficulties, it’s worth noting that saliva stands out as the optimal specimen for analysis due to its non-invasive nature and cost-effectiveness for patients.

Overall, our findings emphasize the importance of CD166 expression as a possible diagnostic marker in the context of BI-RADS assessment, adding to our expanding awareness of its potential utility in the early diagnosis and monitoring of breast cancer. A large number of samples and multi-center studies are needed to properly understand the clinical significance of these findings and to establish CD166 as a viable addition to breast cancer detection.

## Conclusions

This study undertook the essential task of elucidating the relationship between CD166 expression levels and the well-established BI-RADS grading system. By delving into this association, the aim was to determine whether CD166 could serve as an adjunct biomarker augmenting the predictive capacity of BI-RADS. The study’s findings suggest a significant difference in CD166 expression between breast cancer patients and control volunteers, which serves as an informative biomarker across distinct BI-RADS categories. This distinction could serve as a valuable complementary test for diagnosing breast abnormalities.
